# Supplementary material for: Genomic Analyses Implicate the Amazon–Orinoco Plume as the Driver of Cryptic Speciation in a Swimming Crab
Source: Genes (Basel). 2022 Dec 1;13(12):2263. doi: 10.3390/genes13122263 (PMC9777557; doi:10.3390/genes13122263)
Supplement: Supplementary file 1 [file genes-13-02263-s001.zip › genes-2000298-supplementary.pdf]

SUPPORTING INFORMATION

Genomic Analyses Implicate the Amazon–Orinoco Plume as the Driver of Cryptic Speciation in a Swimming Crab

Pedro A. Peres [1,2,*], Heather Bracken-Grissom [1,3], Laura E. Timm [4,5] and Fernando L. Mantelatto [2]

[1] Institute of Environment and Department of Biology, Florida International University (FIU), Miami, FL 33199, USA
[2.] Laboratory of Bioecology and Systematics of Crustaceans (LBSC), Faculty of Philosophy, Sciences and Letters at Ribeirão Preto (FFCLRP), University of São Paulo (USP), Ribeirão Preto, SP 14040-901, Brazil
[3] Department of Invertebrate Zoology, National Museum of Natural History-Smithsonian, Washington, WA 20013-7012, USA
[4] College of Fisheries and Ocean Sciences, University of Alaska Fairbanks, Fairbanks, AK 99775, USA
[5] Auke Bay Laboratories, Alaska Fisheries Science Center, NOAA National Marine Fisheries Service, Juneau, AK 99801, USA
* Correspondence: pedro.peres27@gmail.com

Table S1. Individuals of *Callinectes ornatus* analyzed in COI and ddRAD analyses. Vouch. Coll.: Voucher Collection ID. COI and GB COI: indicates if the specimen was used in COI analyses with the accession number. ddRAD: indicates all individuals used (total) and included in the analyses after filtering steps (final dataset). Locality indicates where they were sampled. Latitude and Longitude are showed as indicated on the original Voucher Collection ID tag or GB, otherwise we indicated as not available (n/a). CCDB = Crustacean Collection of Department of Biology, FFCLRP/USP, Brazil; FLMNH = Florida Museum of Natural; HBG = Florida International Crustacean Collection; ULLZ = University of Louisiana at Lafayette Zoological Collection; MNHN = Muséum National D'Histoire Naturelle; USNM = National Museum of Natural History.

Vouch. Coll.	COI	GB COI	ddRAD (total)	ddRAD (final dataset)	Locality	Latitude	Longitude
CCDB 0126	X	MT272165	X	X	Ubatuba, São Paulo, Brazil	23° 26' 33"S	45°03'8"W
CCDB 0126	X	MT272160	X	X	Ubatuba, São Paulo, Brazil	23° 26' 33"S	45°03'8"W
CCDB 0126			X	X	Ubatuba, São Paulo, Brazil	23° 26' 33"S	45°03'8"W
CCDB 1537	X	MT272164	X	X	Ubatuba, São Paulo, Brazil	23° 28' 31" S	44° 57' 18" W
CCDB 1537			X	X	Ubatuba, São Paulo, Brazil	23° 28' 31" S	44° 57' 18" W
CCDB 1537			X	X	Ubatuba, São Paulo, Brazil	23° 28' 31" S	44° 57' 18" W
CCDB 351	X	MT272134	X		Ubatuba, São Paulo, Brazil	23°26'10"S	45°01'36"W
CCDB 352	X	MT272168			Ubatuba, São Paulo, Brazil	23° 26' 10" S	45° 01' 36" W
CCDB 353			X	X	Ubatuba, São Paulo, Brazil	23° 26' 10" S	45° 01' 36" W
CCDB 354			X	X	Ubatuba, São Paulo, Brazil	23° 26' 10" S	45° 01' 36" W
CCDB 355	X	MT272159	X	X	Ubatuba, São Paulo, Brazil	23° 26' 10" S	45° 01' 36" W
CCDB 356	X	MT272167	X	X	Ubatuba, São Paulo, Brazil	23° 26' 10" S	45° 01' 36" W
CCDB 357	X	MT272147	X		Ubatuba, São Paulo, Brazil	23° 26' 10" S	45° 01' 36" W
CCDB 358	X	MT272135	X	X	Ubatuba, São Paulo, Brazil	23° 26' 10" S	45° 01' 36" W
CCDB 359	X	MT272136	X	X	Ubatuba, São Paulo, Brazil	23° 26' 10" S	45° 01' 36" W
CCDB 3929	X	MT272170	X	X	Rio Grande, Rio Grande do Sul, Brazil	32º 10' 23" S	52º 06' 10" W
CCDB 3929	X	MT272155	X	X	Rio Grande, Rio Grande do Sul, Brazil	32º 10' 23" S	52º 06' 10" W
CCDB 3929	X	MT272149	X	X	Rio Grande, Rio Grande do Sul, Brazil	32º 10' 23" S	52º 06' 10" W
CCDB 3929	X	MT272142	X	X	Rio Grande, Rio Grande do Sul, Brazil	32º 10' 23" S	52º 06' 10" W
CCDB 3929	X	MT272156	X	X	Rio Grande, Rio Grande do Sul, Brazil	32º 10' 23" S	52º 06' 10" W
CCDB 3929	X	MT272157	X	X	Rio Grande, Rio Grande do Sul, Brazil	32º 10' 23" S	52º 06' 10" W
CCDB 3929	X	MT272150	X	X	Rio Grande, Rio Grande do Sul, Brazil	32º 10' 23" S	52º 06' 10" W
CCDB 3929	X	MT272143	X	X	Rio Grande, Rio Grande do Sul, Brazil	32º 10' 23" S	52º 06' 10" W
CCDB 3929	X	MT272144	X	X	Rio Grande, Rio Grande do Sul, Brazil	32º 10' 23" S	52º 06' 10" W

CCDB 3929	X	MT272166	X	X	Rio Grande, Rio Grande do Sul, Brazil	32º 10' 23" S	52º 06' 10" W
CCDB 3929	X	MT272145	X	X	Rio Grande, Rio Grande do Sul, Brazil	32º 10' 23" S	52º 06' 10" W
CCDB 3929			X		Rio Grande, Rio Grande do Sul, Brazil	32º 10' 23" S	52º 06' 10" W
CCDB 3929			X		Rio Grande, Rio Grande do Sul, Brazil	32º 10' 23" S	52º 06' 10" W
CCDB 3929			X		Rio Grande, Rio Grande do Sul, Brazil	32º 10' 23" S	52º 06' 10" W
CCDB 3929			X	X	Rio Grande, Rio Grande do Sul, Brazil	32º 10' 23" S	52º 06' 10" W
CCDB 3929			X	X	Rio Grande, Rio Grande do Sul, Brazil	32º 10' 23" S	52º 06' 10" W
CCDB 3929			X	X	Rio Grande, Rio Grande do Sul, Brazil	32º 10' 23" S	52º 06' 10" W
CCDB 4056	X	MT272137	X	X	Vitória, Espírito Santo, Brazil	28° 18' 8" S	40° 17' 8" W
CCDB 4056	X	MT272138	X	X	Vitória, Espírito Santo, Brazil	28° 18' 8" S	40° 17' 8" W
CCDB 4056	X	MT272169	X	X	Vitória, Espírito Santo, Brazil	28° 18' 8" S	40° 17' 8" W
CCDB 4251	X	MT272152	X	X	Macaé, Rio de Janeiro, Brazil	22° 25' 1" S	41° 44' 5" W
CCDB 4401	X	MT272151	X	X	Camboriú, Santa Catarina, Brazil	27º 00' 03" S	48º 37' 10" W
CCDB 5421	X	MT272132	X	X	Baia Formosa, Rio Grande do Norte, Brazil	6º 21' 11" S	35º 00' 1" W
CCDB 5421	X	MT272146	X	X	Baia Formosa, Rio Grande do Norte, Brazil	6º 21' 11" S	35º 00' 1" W
CCDB 5421	X	MT272133	X	X	Baia Formosa, Rio Grande do Norte, Brazil	6º 21' 11" S	35º 00' 1" W
CCDB 5421			X		Baia Formosa, Rio Grande do Norte, Brazil	6º 21' 11" S	35º 00' 1" W
CCDB 5421			X	X	Baia Formosa, Rio Grande do Norte, Brazil	6º 21' 11" S	35º 00' 1" W
CCDB 6105	X	KY940161	X	X	Parnamirim, Rio Grande do Norte, Brazil	5° 58' S	35° 07" W
CCDB 6105	X	MT272139	X		Parnamirim, Rio Grande do Norte, Brazil	5° 58' S	35° 07" W
CCDB 6130	X	MT272154	X	X	Aracajú, Sergipe, Brazil	11° 00' 70" S	37° 03' 06" W
CCDB 6130	X	MT272161	X	X	Aracajú, Sergipe, Brazil	11° 00' 70" S	37° 03' 06" W
CCDB 6130	X	MT272153	X	X	Aracajú, Sergipe, Brazil	11° 00' 70" S	37° 03' 06" W
CCDB 6130	X	MT272148	X	X	Aracajú, Sergipe, Brazil	11° 00' 70" S	37° 03' 06" W
CCDB 6130	X	MT272140	X	X	Aracajú, Sergipe, Brazil	11° 00' 70" S	37° 03' 06" W
CCDB 6130	X	MT272162	X	X	Aracajú, Sergipe, Brazil	11° 00' 70" S	37° 03' 06" W
CCDB 6130	X	MT272163	X	X	Aracajú, Sergipe, Brazil	11° 00' 70" S	37° 03' 06" W
CCDB 6130	X	MT272141	X	X	Aracajú, Sergipe, Brazil	11° 00' 70" S	37° 03' 06" W
FLMNH 11249	X	MT272175	X		Trinidad Island, Trinidad & Tobago	n/a	n/a
FLMNH 11409(2)	X	MT272178	X	X	Florida Keys, Florida, USA	24° 40' 22" N	81° 14' 26" W
FLMNH 11409(3)	X	MT272180	X		Florida Keys, Florida, USA	24° 40' 22" N	81° 14' 26" W
FLMNH 11409(4)	X	MT272173	X		Florida Keys, Florida, USA	24° 40' 22" N	81° 14' 26" W
FLMNH 1476(1)	X	MT272181	X	X	Cape Sable, Florida, USA	25° 02" N	81° 20" W
FLMNH 1476(2)	X	MT272172	X		Cape Sable, Florida, USA	25° 2" N	81° 20" W
FLMNH 19804	X	MT272174	X	X	Florida Keys, Florida, USA	24° 31' 02" N	81° 58' 21" W
FLMNH 26242	X	MT272179	X	X	Biscayne Bay, Florida, USA	25° 27' 06" N	80° 11' 49" W

FLMNH 32103	X	MT272158	X	X	Saint Martin	18° 6' 14" N	63° 14' 25" W
FLMNH 34910	X	MT272176			Indian River Lagoon, Florida, USA	29° 43' 02" N	81° 14' 49" W
FLMNH 34910	X	MT272171	X	X	Indian River Lagoon, Florida, USA	29° 43' 02" N	81° 14' 49" W
FLMNH 3982	X	MT272177	X	X	Dry Tortugas, Florida, USA	24° 26' 54" N	82° 17' 21" W
HBG 1026			X	X	Summerland Key, Florida, USA	n/a	n/a
HBG 9815			X	X	Summerland Key, Florida, USA	n/a	n/a
LCD 2965	X	KY940189			Recife, Pernambuco, USA	n/a	n/a
UF 11249	X	KY94024			Trinidad Island, Trinidad & Tobago	n/a	n/a
ULLZ 4178	X	MF490074			Ubatuba, São Paulo, Brazil	23° 26' 41"S	45° 2' 40"W
MNHN-IU-2013-18523	X	MN811215			Ilet la Mere, French Guiana	4° 53' 17.5776" N	52° 10' 2.1864" W
MNHN-IU-2014-10394	X	MN811213			Ile Saint-Joseph, French Guiana	5° 16' 58.8612" N	52° 35' 6.3528" W
MNHN-IU-2014-8208	X	MN811214			Ile Saint-Joseph, French Guiana	5° 16' 59.16" N	52° 35' 6.0648" W
USNM:IZ:1446092	X	MK308023			Morehead City Channel, North Carolina, USA	34° 43' 21.36" N	76° 41' 13.2" W
USNM:IZ:1446094	X	MK308109			Morehead City Channel, North Carolina, USA	34° 43' 21.36" N	76° 41' 13.2" W
USNM:IZ:1446099	X	MK308332			Beaufort Channel, North Carolina, USA	34° 42' 50.76" N	76° 40' 47.28" W
USNM:IZ:1446366	X	MK308212			Beaufort Channel, North Carolina, USA	34° 43' 21.72" N	76° 41' 13.2" W
USNM:IZ:1446367	X	MK308119			Beaufort Channel, North Carolina, USA	34° 43' 21.72" N	76° 41' 13.2" W
USNM:IZ:1446368	X	MK308186			Beaufort Channel, North Carolina, USA	34° 43' 21.72" N	76° 41' 13.2" W
CCDB6667	X	OP817087			São José do Ribamar, Maranhão, Brazil	02° 29' 49" S	44° 02' 04" W
CCDB6667	X	OP817088			São José do Ribamar, Maranhão, Brazil	02° 29' 49" S	44° 02' 04" W
CCDB6667	X	OP817089			São José do Ribamar, Maranhão, Brazil	02° 29' 49" S	44° 02' 04" W
CCDB6667	X	OP817086			São José do Ribamar, Maranhão, Brazil	02° 29' 49" S	44° 02' 04" W
CCDB6667	X	OP917090			São José do Ribamar, Maranhão, Brazil	02° 29' 49" S	44° 02' 04" W
